# Osteosarcoma in Adult Patients Living with HIV/AIDS

**DOI:** 10.1155/2013/219369

**Published:** 2013-03-14

**Authors:** Leonard C. Marais, Nando Ferreira

**Affiliations:** Tumour, Sepsis and Reconstruction Unit, Grey's Hospital, Townbush Road, Pietermaritzburg 3201, KwaZulu-Natal, South Africa

## Abstract

*Background*. HIV infection has reached epidemic proportions in South Africa, with an estimated prevalence of 21.5% in adults living in the province of KwaZulu-Natal. Several malignancies have been identified as part of the spectrum of immunosuppression-related manifestations of HIV infection. Very few reports, however, exist regarding the occurrence of non-AIDS-defining sarcomas in the extremities or limb girdles. *Methods*. A retrospective review was performed on all adult patients, between the ages of 30 and 60 years, with histologically confirmed osteosarcomas of the appendicular skeleton referred to a tertiary-level orthopaedic oncology unit. *Results*. Five out of the nine patients (62.5%) included in the study were found to be HIV positive. The average CD_4_ count of these patients was 278 (237–301) cells/mm^3^, indicating advanced immunological compromise. Three of the malignancies in HIV-positive patients occurred in preexisting benign or low-grade tumours. *Conclusion*. A heightened index of suspicion is required in HIV patients presenting with unexplained bone and joint pain or swelling. Judicious use of appropriate radiological investigation, including magnetic resonance imaging of suspicious lesions and timely referral to an appropriate specialized orthopaedic oncology unit, is recommended.

## 1. Introduction

The prevalence of human immunodeficiency virus (HIV) infection in Southern Africa has reached epidemic proportions. Midyear estimates published by Statistics South Africa estimates the national prevalence of HIV infection in adults at 10.6% [1]. In the province of KwaZulu-Natal the situation is worse, with an estimated 21.5% of resident adults between the ages of 15 and 49 years being infected with HIV [2].

Several malignancies have been identified as part of the spectrum of immunosuppression-related manifestations of HIV infection. Principle amongst these is the AIDS-defining cancers, including Kaposi Sarcoma, non-Hodgkin's lymphoma, and cervical cancer. Several long-term studies have indicated an increased risk for the development of a multitude of non-AIDS-defining cancers in HIV/AIDS patients [3–10]. These comprehensive long-term follow-up studies rarely mention the occurrence of bone and soft tissue tumours and also fail to describe the histological nature and site of these malignancies. The increased incidence of EBV-associated smooth muscle tumours, in patients living with HIV, has previously been described [11]. Most of these tumours were, however, found in the trunk, head, or neck. Very few reports exist on the occurrence of non-AIDS-defining sarcomas in the extremities or limb-girdles [12]. The occurrence of osteosarcoma, specifically, has not previously been described.

Osteosarcoma is the most common primary malignancy involving bone, when myeloma is excluded. Nonetheless, it is rare, representing less than 1% of all cancers diagnosed in the United States of America. Due to its relative rarity, with an annual incidence of approximately four per million population, osteosarcoma has been classified as an orphan disease by the WHO. Conventional osteosarcoma is largely a disease of the young, with a second peak of incidence in the elderly [13]. SEER data indicated an annual incidence, for patients younger than twenty-five and older than sixty of 4.4 and 4.2 per million population, respectively. Conversely the incidence rate in patients between to ages of twenty-five and sixty was found to be only 1.7 per million per annum [14]. The development of conventional osteosarcoma in a patient between the ages of thirty and sixty can therefore be seen as an extremely rare event.

A comprehensive literature review revealed no publications regarding the occurrence of osteosarcoma in people living with HIV. The aim of this retrospective study is to determine if an association exists between the development of osteosarcoma and HIV infection in adult patients.

## 2. Methods and Materials

A retrospective review was performed on all adult patients, between the ages of 30 and 60 years, with osteosarcomas of the appendicular skeleton. These patients were all referred to a tertiary-level orthopaedic oncology unit, over the 3-year period between July 2009 and July 2012. All patients with histologically confirmed sarcomas of the extremity or limb girdles were included in the study. Histology was obtained by formal incisional biopsy in all cases. Diagnosis was subsequently confirmed by combined radiological and histological evaluation.

Exclusion criteria were patients younger than 30 years and older than 60 years of age, soft tissue osteosarcoma, patients whose HIV status were not known, and cases where the records were insufficient with regard to the required information. 

The patient's charts were subsequently reviewed and data extracted in order to describe the patient demographics, HIV status, CD_4_ count and use of antiretroviral medication, onset of symptoms, pathology, and presence of metastasis. The management strategy was also noted. Systemic staging involved standard laboratory investigations, CT scanning of the patient's chest and abdomen, and a technetium bone scintigraphy.

Ethical approval for this study was obtained from the UMgungundlovu Health Ethics Review Board.

## 3. Results

A total of 11 patients, between the ages of 30 and 60 years, were identified with skeletal osteosarcoma. One HIV-positive patient and one HIV-negative patient absconded from hospital and were lost to followup. One patient, with an unknown HIV status, passed away prior to biopsy and was also excluded from the study population. Eight patients met the inclusion criteria. The average age was 42 years, ranging from 30 to 54 years. Five patients were HIV positive, with an average CD_4_ count of 278 cells/mm^3^ (range 237–301 cells/mm^3^). The average age of the HIV-positive patients was 41 years (range from 30 to 51 years). Only one of the HIV-positive patients was on antiretroviral treatment at the time of diagnosis (Table 1).

### 3.1. Clinical Presentation

Metaphyseal involvement around the knee joint was the most commonly affected site, with three tumours located in both the distal femur and proximal tibia.

The proximal femur and proximal humerus were the second most common sites, with one lesion each. The time to presentation varied significantly, with patient's symptoms being present for between two and 60 months (mean seven months) prior to referral to the orthopaedic oncology unit. The mean time to presentation in HIV-positive patients was 12 months.

### 3.2. Histology

**Figure 1:**
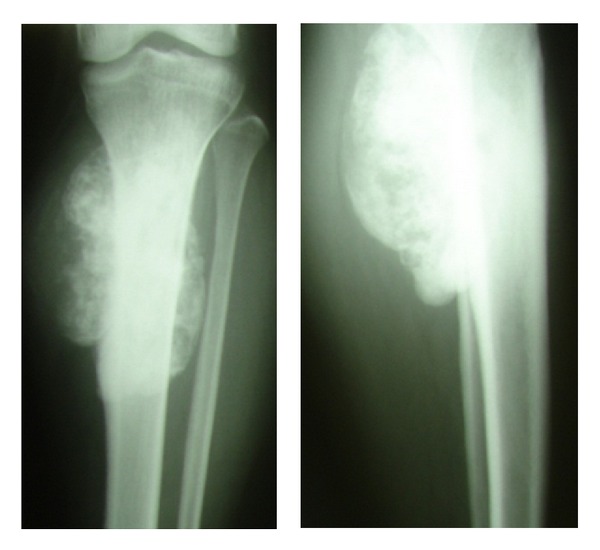
Anterior-posterior and lateral radiographs of the proximal tibia showing a sclerotic lesion suggestive of parosteal osteosarcoma.

**Figure 2:**
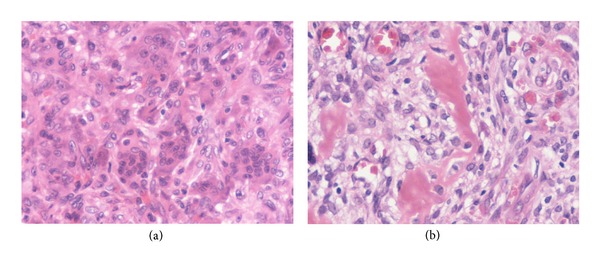
Conventional osteosarcoma (b) within a preexisting histological confirmed giant cell tumour (a), suggesting primary malignant transformation.

Two HIV-negative patients were diagnosed with conventional osteosarcoma, while the third was diagnosed with telangiectatic osteosarcoma. Histological evaluation of the tumours in HIV-positive patients revealed two conventional osteosarcomas and two high grade surface lesions. Radiological evaluation of the high-grade surface lesions suggested that these tumours developed in preexisting parosteal osteosarcomas (Figure 1). The final osteosarcoma in the HIV-positive group developed in a preexisting, histologically confirmed, giant cell tumour. This patient did not receive radiotherapy prior to diagnosis (Figure 2).

### 3.3. Prognostic Factors

Systemic staging revealed multiple lung metastases in four of the five HIV-positive patients. One patient had skeletal metastasis in conjunction with pulmonary metastasis. Two HIV-negative patients had one or more pulmonary metastases at time of presentation, while one patient had no systemic spread. Serum alkaline phosphatase (ALP) and lactate dehydrogenase (LDH) levels were found to be comparable in both groups, with an average ALP of 248 IU/L and LDH of 1066 IU/L in the HIV-positive group.

### 3.4. Management

**Figure 3:**
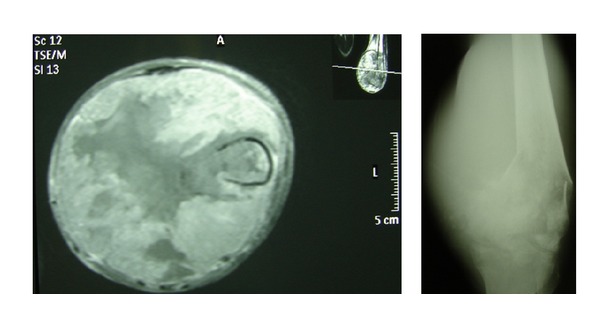
Pathological femur fracture as a result of advanced local disease.

**Figure 4:**
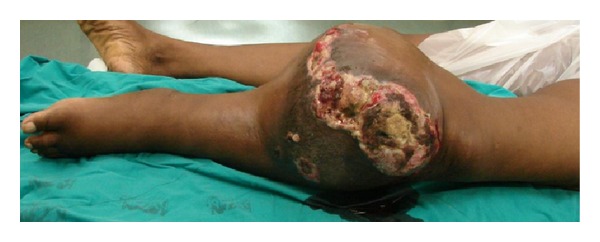
Massive ulcerating osteosarcoma necessitating palliative amputation.

Four of the five HIV-positive patients were treated with palliative amputation and chemotherapy in order to achieve adequate pain control and enable mobilization of the patient. One tumour, originating in the proximal humerus, was considered inoperable and was treated with radiotherapy and palliative chemotherapy. Two HIV-negative patients were treated with amputation and adjuvant chemotherapy. The first of these patients had abandoned therapy in a different province and presented to our unit approximately 6 months following onset of symptoms with extensive local progression and a pathological fracture of his femur (Figure 3). The second HIV-negative patient presented with a massive ulcerating tumour (Figure 4). The third patient refused surgery and was offered palliative radio- and chemotherapy.

## 4. Discussion

An association between mesenchymal malignancies and HIV infection has not clearly been established. Despite several large, long-term studies identifying the occurrence of bone and soft tissue tumours in HIV-positive patients, the relationship with osteosarcoma remains unsure.

Osteosarcoma occurs extremely rarely in adults between the ages of 30 and 60 years. South Africa currently lacks a population-based comprehensive cancer register. The most recent statistics was released in 2004, recording a total of 198 bone tumours. The specific national incidence of osteosarcoma has not been published. A previous report, from the same orthopaedic oncology unit, noted a total of 25 cases of osteosarcoma (in all age groups) over a 2-year period [15].

HIV infection is, however, extremely common in this age group in KwaZulu-Natal, with an estimated 21.5% of adults between the ages of 15 and 50 years being infected [2]. This retrospective review revealed that 62.5% of patients between the ages of 30 and 60 years with biopsy confirmed osteosarcoma were HIV positive, suggesting a trend towards increased incidence of osteosarcoma in patients living with HIV. All of the HIV-positive patients with concurrent osteosarcoma fulfilled the WHO immunological criteria for advanced HIV infection with a CD_4_ count of less than 350 cells/mm^3^ [16]. Larger prospective studies are required to investigate the possibility that osteosarcoma either results in a decrease in the patient's immune competency or, alternatively, if osteosarcoma tends to occur in patients with immunologically advanced HIV infection. The relatively long interval in this series between onset of symptoms and presentation to a specialized unit (mean six months) further complicates interpretation of the CD_4_ count data.

No significant difference, in terms of patient demographics, was found between HIV-positive and -negative patients. The occurrence of two high-grade surface lesions in the HIV-positive group is, however, of particular interest. Osteosarcomas occurring on the surface of a bone are typically low-or intermediate-grade tumours. High-grade surface lesions are exceedingly rare, comprising approximately 8% of surface lesion and approximately 1% of all osteosarcomas [17, 18]. To further illustrate the rarity of this subtype of osteosarcoma, it should be noted that the Japanese Foundation for Cancer Research recorded only four cases over a 27-year period between 1978 and 2005 [19]. It may be argued that HIV-associated immune incompetence may possibly have resulted in dedifferention of preexisting low-grade surface lesions. This assumption is supported by the fact that one of the other high-grade osteosarcomas in the HIV-positive group was histologically confirmed to have occurred in another preexisting tumour (giant cell tumour). Radiological evaluation of the high-grade surface lesions in this study suggested that these patients both had a preexisting low grade parosteal osteosarcoma.

LDH and the presence of metastases have previously been shown to be important factors in determining the prognosis of a patient diagnosed with osteosarcoma [20]. No significant difference could be shown with relation to the prognosis between HIV-positive and -negative patients, and the small sample size prohibited drawing any firm conclusions in this regard.

The immunological basis of the development of malignancies in HIV-infected individuals is not yet completely understood. The pathogenesis of many malignancies associated with HIV infection involves concomitant viral infection. B-cell non-Hodgkin's Lymphoma in HIV patients has been shown to be associated with the Epstein-Barr virus and Human herpes virus-8 (HHV-8) infection [21]. HIV, in itself, may be involved in carcinogenesis through direct and indirect mechanism. A direct role of HIV in the development of the Kaposi sarcoma has been proposed, where the virus is thought to promote cellular growth through the HIV-1 tat protein [22]. One direct mechanism whereby retroviruses can cause cancer is through oncogene transduction. This mechanism has, however, only been seen in simple retroviruses and not with complex retroviruses like HIV. Many retroviruses do not possess viral oncogenes, but initiate tumour formation through the integration of proviral DNA near normal cellular proto-oncogenes, thereby activating their expression, by a mechanism termed proviral insertional mutagenesis. Expression of *c-myc *proto-oncogene has been shown to be induced during HIV infection [23]. Activation of *c-myc *oncogene has also been described in AIDS-associated lymphoma [24]. Furthermore the importance of the *c-myc* oncogene has been firmly established in the pathogenesis of osteosarcoma [25]. However, if, indeed, there is an association between HIV infection and osteosarcoma, it is more likely through the indirect mechanism of immunosuppression. 

This series has several shortcomings. The small sample size makes definitive conclusions impossible. Larger studies, preferably in the form of national cancer registries, would be required to confirm the suspected association between HIV infection and osteosarcoma. Due to the rural nature of the region where this study was performed, long-term followup was inadequate. A prospective study of HIV-infected patients with osteosarcoma would be more appropriate in determining the outcome of treatment. 

Despite these shortcomings, the trend towards an increased incidence of osteosarcoma in adult patients with HIV infection warrants increased vigilance. A heightened index of suspicion is required in HIV patients presenting with unexplained bone and joint pain or swelling. Judicious use of appropriate radiological investigation, including magnetic resonance imaging of suspicious cases and timely referral to a specialized orthopaedic oncology unit, is recommended.

## Figures and Tables

**Table 1:** Clinical and pathological features of patients included in the study.

Patient	Age	HIV status	CD_4_ count	Duration of symptoms (months)	Preexisting lesion	Diagnosis	Location of tumour	LDH	Metastasis	Management
1	30	+	292	12	Parosteal osteosarcoma	High-grade surface osteosarcoma	Proximal tibia	675	None	Amputation, adjuvant chemotherapy
2	40	+	274	2	Parosteal osteosarcoma	High-grade surface osteosarcoma	Distal femur	383	Lung	Palliative amputation and chemotherapy
3	51	+	288	6	n/a	Conventional osteosarcoma	Distal femur	854	Lung, bone	Palliative amputation and chemotherapy
4	37	+	301	24	Giant cell tumour	Conventional osteosarcoma	Proximal tibia	441	Lung	Palliative amputation and chemotherapy
5	50	+	237	60	n/a	Conventional osteosarcoma	Proximal humerus	2977	Lung	Palliative chemo- and radiotherapy
6	43	−	n/a	7	n/a	Conventional osteosarcoma	Proximal tibia	947	Lung	Palliative amputation and chemotherapy
7	33	−	n/a	6	n/a	Conventional osteosarcoma	Distal femur	1139	Lung	Palliative amputation and chemotherapy
8	44	−	n/a	7	n/a	Telangiectatic osteosarcoma	Proximal femur	415	None	Refused surgery, chemo- and radiotherapy
